# Neural Mechanisms Underlying the Dynamic Updating of Native Language

**DOI:** 10.1162/nol_a_00023

**Published:** 2020-11-01

**Authors:** Kelly Sharer, Malathi Thothathiri

**Affiliations:** Department of Speech, Language and Hearing Sciences, The George Washington University, Washington, DC, USA; Department of Speech, Language and Hearing Sciences, The George Washington University, Washington, DC, USA

**Keywords:** sentence comprehension, syntax, adaptation, left inferior frontal gyrus (LIFG), multiple-demand (MD) network, cognitive control

## Abstract

Language users encounter different sentence structures from different people in different contexts. Although syntactic variability and adults’ ability to adapt to it are both widely acknowledged, the relevant mechanisms and neural substrates are unknown. We hypothesized that syntactic updating might rely on cognitive control, which can help detect and resolve mismatch between prior linguistic expectations and new language experiences that countervail those expectations and thereby assist in accurately encoding new input. Using functional neuroimaging (fMRI), we investigated updating in garden-path sentence comprehension to test the prediction that regions within the left inferior frontal cortex might be relevant neural substrates, and additionally, explored the role of regions within the multiple demand network. Participants read ambiguous and unambiguous main-verb and relative-clause sentences. Ambiguous relative-clause sentences led to a garden-path effect in the left pars opercularis within the lateral frontal cortex and the left anterior insula/frontal operculum within the multiple demand network. This effect decreased upon repeated exposure to relative-clause sentences, consistent with updating. The two regions showed several contrastive patterns, including different activation relative to baseline, correlation with performance in a cognitive control task (the Stroop task), and verb-specificity versus generality in adaptation. Together, these results offer new insight into how the brain updates native language. They demonstrate the involvement of left frontal brain regions in helping the language system adjust to new experiences, with different areas playing distinct functional roles.

## INTRODUCTION

Humans begin to learn and appear to master the fundamental aspects of their native language during childhood (e.g., Fernald, Thorpe, & Marchman, 2010; Gómez, 2002; Mehler et al., 1988; Newport, 1990; Scott & Fisher, 2009; Smith & Yu, 2008; Werker & Tees, 2005). But they also need to continually adapt this language throughout the lifespan. Adults face considerable variation in how any given language is used—they have to interact with and understand different people who have different lexical and syntactic preferences that vary depending on dialect, education, and social context (Tagliamonte, 2011). In situations where a reader or a listener encounters a distribution of syntactic structures that is different from their own language experience (e.g., a different dialect or register), mechanisms that enable the brain to update syntactic expectations would be advantageous (Fine, Jaeger, Farmer, & Qian, 2013). Observational and experimental evidence suggests that language users are indeed capable of adjusting their native language comprehension in different environments. But the neural mechanisms that support such dynamic updating over the course of syntactic experience are neither known nor fully explored. (For related evidence from priming studies, see Devauchelle, Oppenheim, Rizzi, Dehaene, & Pallier, 2009; Ledoux, Traxler, & Swaab, 2007; Noppeney & Price, 2004; Segaert, Kempen, Petersson, & Hagoort, 2013; Segaert, Menenti, Weber, Petersson, & Hagoort, 2011; Tooley, Traxler, & Swaab, 2009.) The present article seeks to address this question. We develop and test neural predictions arising from an integrative framework that combines ideas from different strands of the neurolinguistics and psycholinguistics literature. To our knowledge, the results offer some of the first neural evidence on syntactic updating.

### Sentence Processing Is Incremental and Sensitive to Syntactic Probabilities

One relevant set of findings and conclusions underlying the current framework pertains to incremental processing within language. Sentences unfold over time. Therefore, listeners and readers could either wait until the end of the sentence to determine its structure and interpretation, or they could use incremental evidence available at any given moment to interpret the sentence on an ongoing basis. The former ensures a correct interpretation but could be inefficient. In contrast, the latter is more efficient but could, on occasion, lead to a wrong interpretation that would have to be corrected.

Considerable prior evidence examining ambiguous, garden-path sentences suggests that sentence processing is incremental. In these types of sentences, a sequence of words is temporarily consistent with multiple syntactic structures. For example, a well-studied ambiguity involves the choice between main verb (MV) and relative clause (RC) structures, as demonstrated in (1) and (2).

(1)  *MV Ambiguous:* The experienced soldiers warned about the dangers before the midnight raid.
(2)  *RC Ambiguous:* The experienced soldiers warned about the dangers conducted the midnight raid.

The initial segments of (1) and (2) are identical and involve the verb *warned*. The two sentences differ in whether this verb is to be ultimately interpreted as the main verb that describes the primary action enacted by the soldiers (who did the warning, as in [1] or as part of a relative clause that provides secondary information about the soldiers (who were warned, as in [2]). Thus, these sentences are temporarily ambiguous between two possible syntactic analyses until the critical disambiguating information (e.g., *conducted the …* in [2]) arrives. Several studies examining this and other syntactic ambiguities have found a garden-path effect whereby unexpected, less common completions (e.g., RC) increase reaction times (RTs), especially during the disambiguating portion of the sentence, as compared to expected, more common completions (e.g., MV; Frazier & Rayner, 1982; MacDonald, 1994; MacDonald, Just, & Carpenter, 1992; Trueswell, Tanenhaus, & Kello, 1993). This suggests that the sentence processing system generates relatively quick syntactic analyses and interpretations that keep pace with (or even jump ahead of) what is heard or read instead of waiting for the end of the sentence and all relevant information (Friederici & Kotz, 2003; Osterhout & Holcomb, 1992; Osterhout, Holcomb, & Swinney, 1994; Trueswell & Tanenhaus, 1994; see also Altmann & Kamide, 1999; Bernolet & Hartsuiker, 2010; Federmeier & Kutas, 1999; Ferretti, McRae, & Hatherell, 2001; Kamide, Altmann, & Haywood, 2003; Tanenhaus, Carlson, & Trueswell, 1989). Note that throughout this paper, we refer to syntactic probabilities, analyses, expectations, and updating for the sake of simplicity. Syntactic probabilities are intertwined with semantic and lexical factors in natural language. Thus, factors other than syntax could guide expectations and interpretations during sentence processing (see, e.g., Kim & Osterhout, 2005; Levy, 2008; Snedeker & Trueswell, 2004; Thothathiri, Kim, Trueswell, & Thompson-Schill, 2012). The present hypotheses about updating expectations are agnostic about whether those expectations arise from syntax alone or from a combination of cues.

Prior evidence suggests that probability is a key factor that drives expectations during sentence processing. Under a probabilistic parsing model (e.g., Hale, 2001; Levy, 2008), multiple possible syntactic structures are activated during incremental sentence processing and preferentially ranked according to their probability derived from past experience. Processing difficulty arises whenever a word is unexpected or surprising given the previous words in the sentence. These accounts can explain the garden-path effects described above: Sentences like (2) lead to processing difficulty in the disambiguating region (e.g., *conducted the …*) because those words are not commonly encountered in the context of the previous words (Hale, 2001; Levy, 2008).

Past evidence also suggests that the brain is sensitive to syntactic probability on different levels, including a verb-general level that tracks how often a structure is experienced independent of the verb, and a verb-specific level that encodes how often a particular verb appears in a particular structure. Sentences that are less common overall lead to greater processing difficulty (Frazier & Rayner, 1982; MacDonald et al., 1992). In addition, the probability distributions used to make predictions during incremental processing can vary according to the verb. For example, Osterhout et al. (1994) demonstrated that reading less common sentential complement (SC) sentences only elicited a P600 (indicating syntactic difficulty and reanalysis) when the sentences contained verbs biased towards an alternative direct-object structure and not when they contained verbs that appeared in SC sentences more frequently (see also Trueswell et al., 1993). In fact, similar verb-specific effects are found even for frequently encountered, less complex structures like the MV. Wilson and Garnsey (2009) reported slower reading times for ambiguous MV sentences containing RC-biased verbs than for those containing MV-biased verbs, indicating the influence of individual verbs’ statistical preferences independent of syntactic complexity.

### Adults Adapt to Changing Syntactic Probabilities

Syntactic probabilities are not static but dynamic, changing with new language input. They also vary depending on modality (e.g., spoken vs. written language), formality (e.g., formal vs. informal speech), the speaker’s dialect, and other factors. Thus, it might be useful for the brain not only to track syntactic probabilities when learning a language, but also to dynamically adapt syntactic expectations in situations where the probabilities differ from prior experience. Emerging evidence suggests that adults do adjust their expectations in this way. For example, one eyetracking study showed that having heard different speakers express different kinds of meanings, listeners came to anticipate the meanings that were most associated with each speaker (Kamide, 2012). Similarly, Kroczek and Gunter (2017) showed that when listeners were exposed to two German speakers, one biased towards using a subject-initial structure and the other biased towards using an object-initial structure, their subsequent parsing of an ambiguous structure varied systematically based on the speaker. This speaker effect was maintained one day after exposure and facilitated quick speaker-based adjustment in a follow-up session nine months later, demonstrating that speaker-dependent updating of syntactic predictions can be maintained over time.

Using a different manipulation, Ryskin and colleagues showed that when exposed to verbs with different biases for different sentence structures, listeners’ interpretations came to be guided by those verb biases, which were manipulated during the course of the experiment (Ryskin, Qi, Duff, & Brown-Schmidt, 2017). More broadly, exposure to uncommon sentence structures appears to facilitate subsequent processing of those sentence structures in both behavioral (Farmer, Fine, Yan, Cheimariou, & Jaeger, 2014; Fine et al., 2013; Wells, Christiansen, Race, Acheson, & MacDonald, 2009) and electrophysiological (Coulson, King, & Kutas, 1998) studies. (Note that there is debate about some behavioral findings and measures—specifically on whether faster reading times could reflect task-level adaptation instead of syntactic updating (Prasad & Linzen, 2019; Stack, James, & Watson, 2018). We address task-based adaptation versus syntactic updating in the Discussion section. For the present, we note that the most recent study on this topic concluded that syntactic updating (going beyond task practice) is detectable if a study is adequately powered (Prasad & Linzen, 2019). In all of these cases, the updated comprehension patterns were observed in adults who were mature native speakers of the language, indicating that the updating of syntactic probabilities continues beyond the acquisition that occurs in childhood.

### Potential Mechanisms: A Hypothesized Role for Cognitive Control

Updating syntactic probabilities minimally requires the brain to encode the frequency of occurrence of different syntactic structures in the current linguistic environment. For example, in a situation where a reader encounters many RC sentences, the brain must (a) know when it is experiencing an RC (and not an MV) structure; and (b) update the probability of an RC such that it becomes relatively more expected in the given situation. In cases of syntactic ambiguity where the actual sentence structure goes against the statistically prevalent pattern in a language, step (a) might require the detection and resolution of conflict between the more expected structure (e.g., MV) and the less expected structure (e.g., RC). Put another way, to update probabilities based on exposure to an RC Ambiguous sentence (like [2] above), the more likely but incorrect MV analysis needs to be discarded in favor of the less likely but correct RC analysis. Following the original proposal of Novick, Trueswell, & Thompson-Schill (2005), growing evidence has corroborated the view that cognitive control—the brain’s ability to regulate behavior and handle conflict between incompatible representations and responses—is a plausible mechanism for such selection between competing alternatives. Behaviorally, cognitive control ability correlates with and causally influences the comprehension of conflict-inducing sentences (Hsu & Novick, 2016; Novick, Kan, Trueswell, & Thompson-Schill, 2009; Thothathiri, Asaro, Hsu, & Novick, 2018). Neurally, similar regions are activated by cognitive control tasks and language processing that involves conflict between incompatible interpretations (Hsu, Jaeggi, & Novick, 2017; January, Trueswell, & Thompson-Schill, 2009; Ye & Zhou, 2009; see the detailed discussion below). Thus, while the precise conditions that necessitate the involvement of cognitive control are sometimes debated, there is near-consensus that this function is relevant for sentence processing, especially when the structure and/or content of the sentence contravenes prior expectations.

The present study extends previous work on cognitive control and the processing of individual garden-path sentences to ask if cognitive control might also be related to how well readers dynamically adapt their syntactic expectations in an environment where such sentences are encountered often. We lay out the specific hypothesis and predictions later after a review of the potential neural substrates below.

### Potential Neural Substrates: A Hypothesized Role for Left Inferior Frontal Cortex Regions

As a starting point, we hypothesized that regions known to be involved in both sentence processing and cognitive control might be plausible substrates for syntactic updating. Areas within the left inferior frontal cortex are obvious candidates because they have been consistently linked to sentence processing in different populations and paradigms. In healthy adults, they show increased activation under higher sentence processing demands (January et al., 2009; Keller, Carpenter, & Just, 2001; Mason, Just, Keller, & Carpenter, 2003; Rodd, Longe, Randall, & Tyler, 2010). In priming paradigms, they show repetition suppression when the sentence structure is repeated (Santi & Grodzinsky, 2010; Segaert et al., 2013; Segaert, Menenti, Weber, Petersson, & Hagoort, 2011; Weber & Indefrey, 2009). Neuropsychological studies further indicate that patients with frontal damage are impaired in sentence comprehension (see, e.g., Novick et al., 2005; Tyler et al., 2011, and references therein). Overall, there is widespread agreement that this patch of cortex is relevant for sentence comprehension.

Beyond this consensus, there are two recurrent issues that are still debated. First, there is anatomical and functional heterogeneity within the left inferior cortex. Many previous studies have suggested explicitly or implicitly that the pars opercularis and pars triangularis are especially relevant for syntactic processing (Ben-Shachar, Hendler, Kahn, Ben-Bashat, & Grodzinsky, 2003; Caplan, Alpert, & Waters, 1998; Caplan, Chen, & Waters, 2008; Grodzinsky & Friederici, 2006; Just, Carpenter, Keller, Eddy, & Thulborn, 1996). However, these findings are also quite variable, leading to some suggestions that the functional segregation might be weak rather than strong (Tyler et al., 2011). Therefore, in the present study, we separately analyzed activation patterns within the pars opercularis, the pars triangularis, and the frontal orbital cortex with the expectation that one or more of those regions would show the predicted effects. We did not take a strong a priori position on which of these subregions might be the most relevant.

Relatedly, the types of operations supported by the different subregions present a second issue. One of the earliest proposals was that different regions were specialized for different aspects of language, such as syntax, phonology, and semantics. For syntax, a longstanding claim is that Broca’s area (roughly BA 44/45) is involved in specific syntactic operations. Support for theories under this umbrella has generally taken the form of showing that higher syntactic complexity leads to greater activation (e.g., Ben-Shachar et al., 2003; Santi & Grodzinsky, 2010). A contrasting perspective has challenged this syntactic specialization view by showing that factors other than syntactic complexity can explain activation within Broca’s area. For example, one study showed that conflict between syntactic and semantic cues explained the activation patterns within this region better than did syntactic structure (e.g., passives vs. actives; Thothathiri et al., 2012). Others have shown that the same Broca’s area subregion within each participant is recruited for nonsentential as well as sentence processing tasks (January et al., 2009; Ye & Zhou, 2009). Such evidence has been used to argue that region(s) within the left frontal cortex support the processing of complex sentence structures via their role in selecting between conflicting representations.

A third class of theories lies between the above two perspectives. For example, the memory, unification, and control model put forward by Hagoort (2016) suggests that the left frontal cortex is involved in unification operations—building larger structures from smaller structures stored in the posterior cortex—but this unification is not limited to syntax. It can also include other domains (e.g., semantics) that require combinatorial processes. At the same time, this model proposes that there might be some spatial specialization such that syntactic, semantic, and phonological unification might especially recruit BA 44/45, BA 45/47, and BA 44/6, respectively (Hagoort, 2016). Thus, under this model, the processes occurring within the left frontal cortex are described in broader terms (e.g., selecting and unifying different representations) that are consistent with the cognitive control account while also allowing for the possibility that those processes might be specialized in a graded way for different representations (e.g., syntactic vs. semantic) in different subregions. Other researchers have proposed that functional specialization (e.g., for syntactic processing) might arise at a network rather than the local level, whereby different networks, all involving a given frontal region, might be important for different tasks (Fedorenko & Thompson-Schill, 2014; Hsu et al., 2017).

Together, extant evidence does not support a strong version of syntactic modularity within the left inferior frontal cortex. Recent accounts that argue for some subspecialization nevertheless reject the notion that parts of the frontal cortex are localized exclusively for syntax (see, e.g., Fedorenko, Blank, Siegelman, & Mineroff, 2020; Fedorenko, Duncan, & Kanwisher, 2012; Hagoort, 2016). They suggest instead that these regions might be engaged in broader linguistic processing and/or unification between different types of representations. But whether there might be specialization for linguistic processing, more broadly, is still debated. Studies that have analyzed individual-specific functional regions of interest (ROIs) have been especially relevant here because group-level analysis can potentially obscure functional differences between neighboring patches of cortex due to variability between subjects. Using subject-specific functional ROIs, Fedorenko et al. (2012) found that nearby clusters within Brodmann areas 44 and 45 had different functional profiles. Within each area, one cluster that was activated more for non-words than sentences showed a domain-general profile, namely activation for the harder versus easier version of a variety of verbal and nonverbal tasks. The other cluster, which was activated more for sentences than non-words, was more selective. It either showed no significant activation or inconsistent results for the non-sentence tasks. The authors concluded that Broca’s area is not homogenous; it contains multiple regions that vary in their degree of generality versus specificity (Fedorenko et al., 2012). A related study showed that the regions activated for sentences over non-words were not significantly activated for math, working memory, and cognitive control tasks (Fedorenko, Behr, & Kanwisher, 2011). Synthesizing such evidence, these researchers have suggested that the left inferior frontal cortex might contain two different kinds of regions—language selective regions that are specifically activated for lexical and combinatorial linguistic processing, and domain-general regions that could be involved in sentence processing, especially with a difficult task, but are not specific to language.

However, other studies using subject-specific functional ROIs have noted significant overlap between sentence comprehension and cognitive control (Hsu et al., 2017; January et al., 2009; Ye & Zhou, 2009). Two studies reported that left frontal regions activated by a cognitive control task (e.g., the Stroop task) were also activated for sentences containing conflict between incompatible interpretations (January et al., 2009; Ye & Zhou, 2009). Hsu et al. (2017) similarly found overlap in the left frontal cortex for Stroop and sentence comprehension (among other tasks). However functional connectivity of the frontal cortex with other brain regions differed for different tasks, supporting a network-based view of specialization.

In the present study, we hypothesize only that cognitive control plays a role in processing and updating the statistics for sentences containing conflict between interpretations (see more below). This claim does not preclude (a) the functional segregation of cognitive control operations for linguistic versus non-linguistic representations (see, e.g., Thothathiri, Gagliardi, & Schwartz, 2012); or (b) the existence of language-specific regions involved in non-conflict-related processes. With respect to (a), several of the studies supporting a role for cognitive control have noted overlap between sentence comprehension and the color-word Stroop task (e.g., January et al., 2009; Hsu & Novick, 2016; Thothathiri et al., 2018). Fedorenko and colleagues too have reported weak Stroop effects in some language-specific functional ROIs in some studies (Fedorenko et al., 2011; Fedorenko et al., 2012). The Stroop task contains linguistic content, so overlap in activation is not inconsistent with some degree of linguistic specialization. With respect to (b), Hsu et al. (2017) note that the claim about cognitive control applies to regions identified using a contrast of conflict versus no-conflict linguistic processing and not to other broader contrasts like sentences versus non-words (which could identify regions engaged in lexical, syntactic, and semantic processes that are unrelated to cognitive control). Using such a conflict-specific approach, they showed that conflict functional ROIs in the left frontal cortex were engaged across syntactic and non-syntactic tasks. We took a similar approach to this and related previous studies in that we examined left inferior frontal activation for conflict-inducing sentences in particular. Additionally, we used behavioral performance in a verbal Stroop task to index individual differences in cognitive control, and asked if those differences predicted individual variation in how activation adapted with exposure. We did not test and therefore do not make claims about the domain generality of the conflict resolution processes (unlike some previous studies). Instead, we focus on whether the cognitive control hypothesis could be extended to longer-term updating going beyond the processing of individual conflict sentences.

### Potential Neural Substrates: Exploratory Investigation of the Multiple Demand Network

The multiple demand (MD) network includes several brain regions in the frontal and parietal cortices associated with domain-general cognitive control (Duncan, 2010, 2013). These regions are sensitive to task difficulty across a wide variety of tasks, including arithmetic, spatial and verbal working memory, and Stroop, demonstrating that their function is not restricted to specific representational domains (Fedorenko, Duncan, & Kanwisher, 2013). The network as a whole has been conceptualized as being important for goal-directed and task-appropriate behavior, including organizing subgoals, directing attention, and selecting task-relevant over irrelevant information (Duncan, 2010, 2013). Individual regions within the network are thought to be important for specific aspects of such behavior. For example, the anterior cingulate cortex (ACC) might be important for detecting and monitoring conflict (Botvinick, Cohen, & Carter, 2004; Ullsperger, Fischer, Nigbur, & Endrass, 2014). The anterior insula and the frontal operculum (AI/FO) might be important for sustained task performance, and the inferior frontal and parietal sulci (IFS and IPS) for faster trial-by-trial adjustments (Dosenbach, Fair, et al., 2007; Dosenbach, Visscher, et al., 2006; Nomura et al., 2010). Given this profile, it is possible that one or more regions within this network, especially those in the frontal cortex, are involved in aiding the processing of conflict-inducing (i.e., RC Ambiguous) sentences that are known to result in processing difficulty. Accordingly, we conducted exploratory analyses within 10 MD ROIs derived from the prior literature (Duncan, 2010).

### An Integrative Framework and Associated Neural Predictions

The hypothesis explored in this paper derives from three features of sentence processing described above and reiterated here:

(a)  Language processing unfolds incrementally as the sentence is heard or read, and this incremental processing is guided by the statistical properties of the language;
(b)  Listeners and readers update their syntactic expectations as the statistical properties of language change with new experiences; and
(c)  Cognitive control is used to handle conflict that arises from the incremental processing of sentences whose structures violate prior expectations and statistical properties.

Based on (a) to (c), we hypothesized that:

*The dynamic updating of native language uses cognitive control, which can help with correctly encoding new language experiences that violate prior expectations and thereby aid in the accurate updating of syntactic probabilities.*

Importantly, we are agnostic as to whether cognitive control plays a direct role in updating probabilities per se. We propose only that one possible (indirect) role of cognitive control is to help the parser arrive at the correct analysis of sentences that violate prior expectations, which in turn serves as the input for updating. Prior behavioral evidence suggests that language users do adapt to the statistics of novel language environments. In this paper, we hypothesized and tested predictions about the neural substrates that are involved in this process.

Based on prior evidence for the involvement of left inferior frontal regions in sentence processing and cognitive control, we predicted that one or more of these regions would be engaged in syntactic updating. As a potential contrast, we also looked at the middle temporal gyrus (MTG), which has been consistently linked to syntactic processing but not cognitive control. Frontal and temporal cortices may be concurrently engaged during sentence processing but play different roles. In particular, it has been suggested that the MTG might subserve the storage of syntactic knowledge while frontal regions might be involved in more dynamic processes, including resolving conflict between or unifying different linguistic representations (e.g., Hagoort, 2016; Novick et al., 2005). Therefore, we predicted that one or more of the pars opercularis, the pars triangularis, and the frontal orbital cortex, but not the MTG, would show patterns corresponding to dynamic adjustment, as outlined below.

Additionally, based on the association between the MD network and domain-general cognitive control, we expected that one or more regions within this network could be involved in handling conflict and/or difficulty during sentence comprehension. Below, we lay out the predictions for the different ROIs after we describe the study design.

### The Present Study

We chose the MV/RC ambiguity described above because it is known to generate robust garden-path effects. Each participant underwent three runs of functional neuroimaging (fMRI). They read ambiguous and unambiguous MV and RC sentences, indicated reading completion, and answered intermittent comprehension questions to verify compliance. There were four types of sentences, (1) to (4), including the first two sentence types repeated from above.

(1)  *MV Ambiguous*: The experienced soldiers warned about the dangers before the midnight raid.
(2)  *RC Ambiguous*: The experienced soldiers warned about the dangers conducted the midnight raid.
(3)  *MV Unambiguous*: The experienced soldiers spoke about the dangers before the midnight raid.
(4)  *RC Unambiguous*: The experienced soldiers who were told about the dangers conducted the midnight raid.

Unambiguous sentences like (3) and (4) served as control conditions for comparison with the corresponding ambiguous sentences (MV Ambiguous was compared to MV Unambiguous, and RC Ambiguous was compared to RC Unambiguous). The unambiguous sentence types led to a single interpretation and were not expected to generate any conflict. Ambiguous sentences like (1) and (2) were temporarily consistent with two possible analyses. We chose verbs that were predominantly biased towards the MV sentence structure (see the Materials and Methods section). This meant that the MV Ambiguous condition was not expected to generate conflict despite the presence of temporary ambiguity, because the expectation created from prior experience would be consistent with the actual sentence structure. In contrast, the RC Ambiguous condition was predicted to involve conflict because prior experience would lead readers to expect MV, which would be contradicted by information in the sentence that indicates an RC structure instead.

The first run preceded the exposure phase of the study. It was intended to evaluate which ROIs showed the garden-path effect, namely increased activation specifically in the case of conflict between the expected and actual sentence structures. Concretely, this corresponds to a larger ambiguity effect for RC sentences (RC Ambiguous minus RC Unambiguous) than MV sentences (MV Ambiguous minus MV Unambiguous). A priori, we predicted that one or more of the left inferior frontal ROIs would show this pattern of activation. Contingent on the results from Run1, we restricted all subsequent analyses to the regions showing the garden-path effect.

The second run constituted the exposure phase and consisted only of RC sentences. We hypothesized that if a region were involved in updating expectations based on new language experiences, it would show a decrease in the RC ambiguity effect from the beginning to the end of the exposure phase. Thus, we would expect a negative slope over the course of the exposure run. Further, we predicted that if updating were related to cognitive control, then individual differences in this ability might correspond to how activation changes with exposure. Specifically, those with better cognitive control could encode the RC experiences more accurately and therefore show larger decreases in the ambiguity effect compared to those with poorer cognitive control. We tested this prediction by computing the correlation between performance on a Stroop task and the change in ambiguity-related activation (slope) from the beginning to the end of the exposure phase.

The third and final run constituted the post-exposure period. It served as a counterpart to the first run, which was pre-exposure. The question here was whether any updating effect observed in the exposure phase persisted across runs, that is to say, over a gap of several minutes and whether this updating was restricted to verbs repeatedly seen in RC sentences or generalized more broadly to verbs not seen during the exposure phase. If updating were verb-specific, we would expect a garden-path effect for unexposed but not exposed verbs and a significant difference between ambiguous RC sentences with the two kinds of verbs. Conversely, if updating were verb-general, we would expect no garden-path effect for either verb type and no difference between ambiguous RC sentences with the two verb types.

Our predictions pertain to each run separately because the three runs had different design characteristics. The exposure phase (Run2) presented verbs in RC structures repeatedly but the other runs did not. MV structures were present in Run1 and Run3, but not Run2. A difference between exposed and unexposed verbs could be investigated in Run3 but not in the other runs. Different design matrices for the three runs could lead to differences in parameter estimates and scale factors. Thus, the results within each run are interpretable but cannot be compared directly across runs (Mumford, 2007; Pernet, 2014).

To summarize, we tested four predictions:

P.1: In Run1, a larger RC than MV ambiguity effect in one or more of the left inferior frontal ROIs.
P.2: In Run2, a negative slope corresponding to a decrease in the RC ambiguity effect from the beginning to the end of the exposure phase in the conflict-sensitive region(s) showing a garden-path effect in Run1.
P.3: In Run2, correlation between Stroop scores and the decrease in ambiguity-related activation over Run2 in the conflict-sensitive regions.
P.4: In Run3, a garden-path effect for unexposed but not exposed verbs in the conflict-sensitive regions, corresponding to verb-specific updating.

In addition to the above main predictions, we explored whether one or more frontal regions within the MD network also showed conflict sensitivity and adaptation over time.

## MATERIALS AND METHODS

### Participants

Thirty-one right-handed and neurotypical native English speakers with normal or corrected to normal vision from the Washington, DC, area participated in the study. Three were excluded due to inability to complete all portions of the fMRI data acquisition, recording equipment malfunction, and falling asleep during the study. Data from the remaining twenty-eight participants (18–27 years; *M* = 21.11; 16 female) were analyzed. All underwent an MRI safety screening and provided informed consent under a protocol approved by the George Washington University Institutional Review Board. Participants were paid $25 or given course credit.

### Materials and Procedure

Participants completed a Stroop task outside the scanner and a sentence reading and baseline task inside the scanner. They were familiarized with each task prior to data acquisition (Stroop: eight practice trials; Sentence reading: eight practice trials (four comprehension questions); Baseline: four practice trials). All stimuli were presented using E-Prime 2.0 (Psychology Software Tools, Pittsburgh, PA). For the Stroop task, participants completed a single iteration of practice. For the scanner tasks (intermixed sentence reading, comprehension questions, and baseline), participants repeated practice two to four times to fully grasp the different response mappings and instructions for the different tasks. Practice ended when a participant achieved greater than 50% accuracy. (Note that for one participant, we could not compute accuracy during practice due to a data recording problem.)

#### The Stroop task

The Stroop task was completed in a quiet testing room outside the MRI scanner. Participants saw one word per trial, displayed in blue, green, or yellow 40-point Courier New font against a black background. They were asked to indicate the font color by pressing the appropriate colored button. Stimuli belonged to four conditions. In the Neutral condition, the words (*deal*, *farmer*, *horse*, *plenty*, *stage*, and *tax*) were unrelated to the font color. The Congruent condition contained words that were the same as the font color (e.g., *blue* in blue font). On Incongruent–Eligible trials, the color words were different from the font color (e.g., *green* in blue font). The word meaning (e.g., green) was an available response option, thereby giving rise to conflict at both the representational and the response levels. In contrast, Incongruent–Ineligible trials consisted of color words (*brown*, *orange*, and *red*) that were different from the font color (e.g., *red* in blue font), but the word meaning (e.g., red) was not an available response option. Therefore, these trials were expected to give rise to representational conflict only (no response conflict).

Participants completed four blocks of 72 trials each (18 neutral, 18 congruent, 18 incongruent–eligible, and 18 incongruent–ineligible trials per block). Each trial began with a 300 ms fixation cross, followed by a 500 ms blank screen and then the stimulus word. Stimuli stayed visible until the participant responded by pressing the 4 (blue), 5 (green), or 6 (yellow) key. Stimulus order was pseudorandomized to avoid more than three repetitions of colors or stimulus words in a row.

#### The sentence reading task

The main sentence reading task was completed inside the scanner. Participants silently read MV and RC sentences presented in black 14-point Courier New font on a white background. When they finished reading a sentence, they used the index finger of their right hand to press the leftmost of three buttons to indicate completion. On some trials, they answered a subsequent yes/no comprehension question by using the middle finger of their right hand to press the middle button for *Yes* or the ring finger of their right hand to press the rightmost button for *No*. The comprehension questions ensured that participants were attending to the task and reading the sentences.

Sentences belonged to one of four types: MV Ambiguous, RC Ambiguous, MV Unambiguous, and RC Unambiguous (see examples [1] to [4] above). Twenty-four sentences were modified from Fine et al. (2013). The remainder were newly created for this study. All verbs used in the ambiguous sentences were at least 1.5 times more likely to appear in MV than RC structures (based on average frequencies calculated from the following corpora: Brown, Wall Street Journal, Switchboard, and British National Corpus; Roland, Dick, & Elman, 2007). Thus, readers were expected to initially interpret the ambiguous sentences as MV, which should lead to garden-pathing when the sentence happens to be RC. The corresponding unambiguous sentence types did not contain any ambiguity. For MV Unambiguous sentences, ambiguity was avoided by using either intransitive verbs (e.g., *remained*) or transitive verbs that have different forms in MV versus RC structures (e.g., *spoke* vs. *spoken*). For RC Unambiguous sentences, ambiguity was avoided by including an explicit relativizer (e.g., *that*, *who*).

Each participant completed three functional runs within the scanner. Run1 and Run3 contained 120 sentences each (30 of each type). Within each of these runs, 30 verbs appeared once in MV Ambiguous and once in RC Ambiguous (a total of 60 ambiguous sentences). Thirty unique verbs occurred in MV Unambiguous sentences, and a different set of 30 unique verbs occurred in RC Unambiguous sentences (a total of 60 unambiguous sentences). Run2 provided exposure to RC sentences. Fifteen of the 30 ambiguous verbs used in Run1 were used eight times each in Run2 (four times in RC Ambiguous and four times in RC Unambiguous. Total *N* = 120). There were no MV sentences. Thus, the design may be summarized as pre-RC-exposure (Run1), RC-exposure (Run2), and post-RC-exposure (Run3).

Twenty sentences in each run were followed by yes/no comprehension questions (e.g., Sentence: *The silly boys who were reprimanded during the play quickly left the auditorium*; Question: *Did the boys leave the auditorium quickly?*; Correct answer: *Yes*. Sentence: *The farm animals washed in their pens at the state fair*; Question: *Were the animals roaming free?*; Correct answer: *No*). The questions were randomly interspersed, followed each sentence type an equal number of times, pertained to different parts of the sentences, and did not call attention to the syntactic ambiguity (when present). This design was chosen to mitigate the possibility of explicit strategies for ambiguity resolution while also encouraging the processing of all parts of each sentence. The correct answer was *No* for half of the trials.

**Figure 1.**
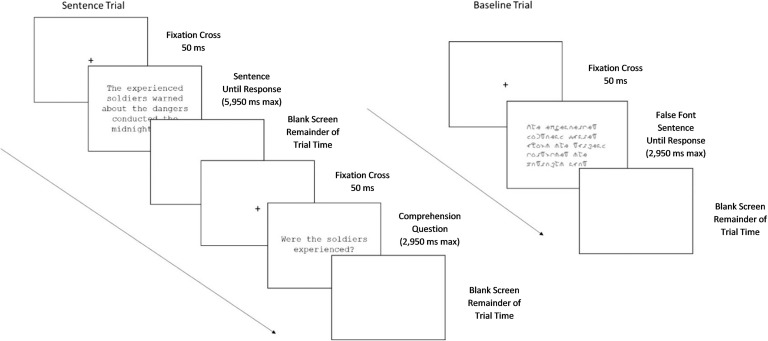
Sequence of stimulus presentation in sentence (left) and baseline (right) trials.

Sentence trials lasted 6 s (50 ms fixation cross followed by sentence presentation for a maximum of 5,950 ms). If a participant responded before the allotted time had finished, a blank screen replaced the stimulus until the trial-time elapsed. Comprehension questions, when present, followed the sentences (after the allotted 6 s). Comprehension question trials lasted 3 s (50 ms fixation cross followed by question presentation for a maximum of 2,950 ms). The question remained on the screen until participants selected a response. Following a response, a blank screen replaced the question until the trial-time elapsed (Figure 1).

Sentence stimuli were counterbalanced in a number of ways. Four counterbalanced lists were created such that any “semantic frame” (e.g., *The experienced soldiers* [*main verb OR relative clause*] *about the dangers* [*preposition OR main verb*] *the midnight raid.*) appeared in an MV Ambiguous structure in one list, an RC Ambiguous structure in another list, and so on. This ensured that the types of events described by the sentences were roughly semantically equivalent between lists. Participants were randomly assigned to a list. We also counterbalanced which verbs were presented in Run2 (verbs presented during Run2 in lists 1 and 3 were absent in lists 2 and 4 and vice versa). Examples illustrating the counterbalancing are shown in the Appendix in the online supporting information located at https://www.mitpressjournals.org/doi/suppl/10.1162/nol_a_00023. Sentence length was adjusted such that there were no significant differences between MV and RC sentences overall (Run1: *t* < 1.5, *p* > 0.05; Run3: *t* < 1, *p* > 0.05).

#### The baseline task

Baseline trials were pseudorandomly interspersed between sentence trials. Participants visually scanned lines of false-font (BACS2serif; Vidal, Chetail, & Content, 2016) and pressed the middle (*Yes*) or the right (*No*) buttons to indicate whether they saw the numeral “4” within the false-font characters. Half of the trials contained the numeral at a randomly selected location within the sequence. The false-font stimuli were matched in the number of lines and angles to a subset of the experimental sentences. Thus, the baseline task controlled for visual processing and manual button presses but did not involve linguistic processing.

Run1 and Run3 each contained 30 baseline trials; Run2 contained 60 trials. The number of baseline trials equaled the number of trials for each sentence type. Thus, the baseline task was not rare, to help minimize the possibility of a “rare event” response that could complicate interpretation (e.g., Busse & Woldorff, 2003). The order of baseline and sentence trials was randomized using Optseq2 (Greve, 2002; http://surfer.nmr.mgh.harvard.edu/optseq/). Each baseline trial lasted 3 s (50 ms fixation cross followed by the stimulus for a maximum of 2,950 ms). If a participant responded before the allotted time had finished, a blank screen replaced the stimulus until the trial-time elapsed (Figure 1).

### Dependent Measures and Analyses

#### Stroop task

Reaction time and accuracy were recorded on each trial. Trials with inaccurate responses were excluded from the RT analysis. For each participant, a normalized representational conflict processing score was calculated as (mean RT on Incongruent–Ineligible trials − mean RT on neutral trials) ÷ (Mean RT on neutral trials) * 100. Higher scores indicate poorer conflict processing. We focused on Incongruent–Ineligible trials and representational conflict because this type of conflict should be more similar to the conflict between alternative interpretations elicited by garden-path sentences (Hsu & Novick, 2016; Thothathiri et al., 2018).

#### Sentence reading fMRI analyses

Structural and functional images were acquired using a 3T Siemens Trio Scanner. All scans took place at the Center for Functional and Molecular Imaging at Georgetown University. A sagittal T1-weighted MPRAGE sequence (TR = 1,900 ms, TE = 2.52 ms, flip angle = 9°, T1 = 900 ms, slice thickness = 1 mm) was used to acquire structural images for each participant. An echoplanar imaging sequence (TR = 3,000 ms, TE = 30 ms, flip angle = 90°, slice thickness = 3 mm) was used to measure the blood oxygen level dependent (BOLD) response during functional imaging.

Image processing and analysis were completed using FSL (Jenkinson, Beckmann, Behrens, Woolrich, & Smith, 2012). Nonbrain voxels were removed and images were LAS-oriented using FLS tools (bet, fslswapdim, and fslorient). Image preprocessing also included motion-correction using MCFLIRT, spatial smoothing using a Gaussian kernel (full width at half maximum = 5 mm), and high-pass filtering (100 Hz). Images were normalized to MNI-152 space. An interleaved slice timing correction was applied to functional activation data. A general linear model containing regressors for each event type, which were convolved with a double gamma hemodynamic response function, was used to analyze each participant’s BOLD response data. For Run1, event types included MV Ambiguous, MV Unambiguous, RC Ambiguous, RC Unambiguous, comprehension questions, and baseline. For Run2, event types included RC Ambiguous, RC Unambiguous, comprehension questions, and baseline. Each of the two sentence event types was modeled separately for the first, second, third, and fourth occurrence of verbs so as to track changes in activation with RC exposure. For Run3, regressors included MV Ambiguous Exposed, MV Ambiguous Unexposed, MV Unambiguous, RC Ambiguous Exposed, RC Ambiguous Unexposed, RC Unambiguous, comprehension questions, and baseline. Standard motion parameters were included.

We analyzed activation in two kinds of ROIs. Participant-specific functional ROIs in the left frontal and temporal cortices were extracted from Run1 as follows. The contrast of all sentences (MV Ambiguous, MV Unambiguous, RC Ambiguous, RC Unambiguous) versus baseline (visual search of false-font characters) in Run1 was subjected to cluster-level error correction (*Z* > 3.1, corrected cluster *p* < 0.05) to obtain all regions that showed more activation for sentences than baseline. Four anatomical regions from the Harvard-Oxford atlas (https://neurovault.org/collections/262/; Desikan et al., 2006) were extracted, three in the left frontal cortex (left pars opercularis = inferior frontal gyrus, pars opercularis, left hemisphere; left pars triangularis = inferior frontal gyrus, pars triangularis, left hemisphere; and left frontal orbital cortex = frontal orbital cortex, left hemisphere) and one in the left temporal cortex (left MTG = posterior middle temporal gyrus, left hemisphere). The functional Sentence > Baseline map was masked with each of the four anatomical regions. For the MTG, we extracted the top 100 voxels showing the most activation for each subject. For the three subregions within the left inferior frontal cortex, to minimize overlap, we extracted the top 30 voxels. Any remaining overlapping voxels were assigned to the label with the highest probability. This procedure yielded functional ROIs in the left frontal and temporal cortices that showed a Sentence > Baseline pattern, across all sentence types (hereafter referred to as Sentence > Baseline ROIs. See Supplementary Figure 1 in the online supporting information).

A second set of 10 group-level functional ROIs from the prior literature that are associated with cognitive demands and thought to be a part of the MD network were defined as 5 mm spheres around the coordinates reported in Duncan (2010). They included lateral frontal, medial frontal and lateral parietal regions (left and right inferior frontal sulcus [IFS] AI/FO, left and right rostrolateral prefrontal cortex, pre-supplementary motor area, dorsal ACC, left and right IPS; hereafter referred to as MD ROIs).

Within these regions, we extracted the median activation for each sentence type relative to baseline for each subject using featquery (FSL). For the Sentence > Baseline ROIs, activation is expected to be significantly above zero by virtue of how the ROIs were selected. However, the critical analyses compared activation between different sentence conditions. These comparisons were orthogonal to the contrast used to identify the ROIs (i.e., the contrast vectors were orthogonal [inner product was zero] and the design was balanced; Kriegeskorte, Simmons, Bellgowan, & Baker, 2009. Further, the key adaptation results are from runs that are distinct from the run used to define the ROIs. The MD ROIs were defined using coordinates from the previous literature—activation for sentences might therefore be either greater or less than baseline. In all cases, repeated measures analyses of variance (ANOVA) were used to determine whether there was a significant ambiguity effect and whether ambiguity effects differed between conditions.

For Run2, we were interested in whether the RC ambiguity effect decreased with exposure. For each subject, we computed the difference in activation between the RC Ambiguous and RC Unambiguous conditions at the first through fourth appearance of the verbs and then used linear regression to obtain the slope of change over time. We used a one-sample *t* test to evaluate whether the mean slope across subjects was less than zero and a binomial test to evaluate whether most participants showed a negative slope.

#### Correlation between neural activation and cognitive control

Skipped Pearson correlations (Pernet, Wilcox, & Rousselet, 2013) were used to evaluate the correlation between each participant’s representational conflict processing score (Stroop) and the change in their RC ambiguity effect with exposure (slope of change within Run2). A significant correlation is one for which the 95% confidence interval does not span zero. Our hypothesis predicts a positive correlation. We expected that participants with better conflict resolution would be more likely to arrive at the correct analysis of RC structures and therefore learn more from RC exposure. Thus, better conflict processing (lower Stroop scores) is predicted to be associated with smaller RC ambiguity effects with increasing exposure (lower, more negative slopes).

It is worth noting that under alternative hypotheses that do not propose a relationship between cognitive control and syntactic ambiguity resolution and updating, the expected correlation could be in the opposite direction. For example, participants with poorer conflict resolution could activate the relevant brain regions more and therefore have a higher likelihood of showing a larger drop in activation over time. In this case, poorer conflict processing (higher Stroop scores) would be associated with lower slopes (a negative correlation). Because our hypothesis explicitly linked cognitive control to correctly encoding new language experiences and updating syntactic probabilities (see An Integrative Framework and Associated Neural Predictions above), we had a principled reason to expect a positive rather than a negative (or non-direction-specific) correlation.

#### Sentence reading behavioral data

In addition to the neural activation data, we collected and analyzed behavioral data in the form of comprehension question accuracy and whole-sentence reading times. The former allowed us to ascertain whether participants were reading and processing different parts of the sentences and thereby verify task compliance.

Whole-sentence reading times were computed from trials where the participants indicated reading completion and answered any accompanying comprehension question correctly. (Trials with no completion response and/or incorrect answers were excluded.) Thus, we restricted the analyses to trials where participants appeared to be following the task instructions. For each participant and run, reading times from the eligible trials were length-adjusted as follows. Linear regression was used to generate a predicted reading time for each sentence based on its length (number of letters). The intercept value was set to 0 (because a sentence with 0 characters would require 0 reading time). Length-adjusted reading times were computed as: (Actual reading time) − (Predicted reading time). A positive number indicates that a sentence was read slower than what would be expected based on length.

We used mixed-effects linear regression (lmer function in R version 1.1.463; Bates, Maechler, Bolker, & Walker, 2015) to evaluate the ambiguity effect and compare the effect between conditions. The models contained all applicable fixed effects (e.g., Ambiguity, Structure), random intercepts, and random slopes. We only simplified the random effects structure when the full model did not converge (described wherever applicable in the Results section).

To evaluate whether the ambiguity effect changed over the course of Run2 and whether that change correlated with Stroop performance, we used the same procedures as for the fMRI analyses. However, our predictions for the behavioral data were weaker than for the neural analyses. The use of whole-sentence rather than word-by-word or segment-by-segment presentation allowed us to examine relatively naturalistic reading. However, this design necessarily meant that the reading times were not restricted to the disambiguating regions within a sentence. They likely indexed the many cognitive operations involved in reading a sentence (Rayner, 1998; Rayner, Kambe, & Duffy, 2000), including those that are unrelated to conflict. One consequence of this could be that the lack of conflict-specificity of the reading time measure makes a correlation with Stroop less likely than in the fMRI analyses. A second consequence could be that the inclusion of non-disambiguating-region processing times in the reading time (e.g., wrap-up effects at the end of a sentence) makes evaluation of the change in the ambiguity effect less precise and predictable.

The critical fMRI analyses, by contrast, focused on regions known to be involved in cognitive control and could therefore prove more sensitive to the component of interest. Accordingly, we opted a priori to focus on neural predictions and therefore discuss the corresponding results at greater length below.

## RESULTS

### fMRI Results

We discuss the main results in the order of the predictions (P.1 to P.4) described above.

#### P.1: Brain regions showing the garden-path effect in Run1

**Figure 2.**
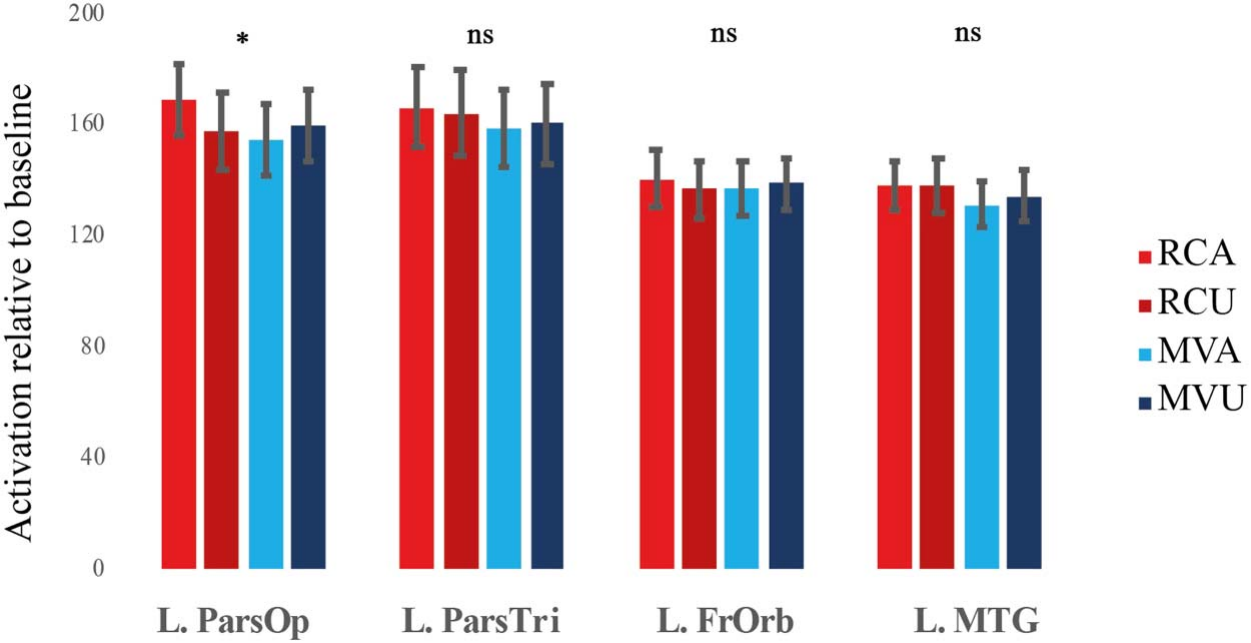
Activation for different sentence types relative to baseline in Run1 within Sentence > Baseline regions of interest. Only the left pars opercularis showed an interaction corresponding to a conflict effect. Here and elsewhere, error bars denote Cousineau-Morey within-subject standard errors (Rmisc package; Morey, 2008). L = left, ParsOp = pars opercularis, ParsTri = pars triangularis, FrOrb = frontal orbital cortex, MTG = middle temporal gyrus, RCA = relative clause ambiguous, RCU = relative clause unambiguous, MVA = main verb ambiguous, MVA = main verb unambiguous.

First, we looked for a pattern of greater ambiguity effect for RC than MV sentences in Run1, which would indicate sensitivity to conflict between the expected and actual sentence structures. We discuss results from the subject-specific left inferior frontal and the MTG ROIs first. The left pars opercularis showed the predicted pattern, namely, a significant interaction between structure and ambiguity, *F*(1, 27) = 10.72, *p* < 0.0125, Bonferroni-corrected for analysis of 4 ROIs. This interaction was due to a significant effect of ambiguity for RC, RC Ambiguous > RC Unambiguous; *F*(1, 27) = 10.72, *p* < 0.05, but not MV, *F*(1, 27) = 2.73, *p* > 0.05, structures. The other frontal regions and the MTG did not show such a conflict effect (Structure × Ambiguity interaction for the left pars triangularis: *F*(1, 27) = 0.51, *p* > 0.05; the left frontal orbital cortex: *F*(1, 27) = 1, *p* > 0.05; and the left MTG: *F*(1, 27) = 0.92, *p* > 0.05). Figure 2 shows the pattern in the left pars opercularis and the other three regions for comparison. The three-way Structure × Ambiguity × Region interaction was significant, Huynh-Feldt corrected *F*(2.59, 69.92) = 3.22, *p* < 0.05, suggesting that the pars opercularis subregion within the left inferior frontal cortex might be especially relevant. Note that analysis of the top 30 voxels in the MTG (paralleling the number of voxels in the frontal ROIs) yielded the same pattern of results. There was no significant Structure × Ambiguity effect in the MTG, *F*(1, 27) = 0.36, *p* > 0.05), and there was a significant three-way Structure × Ambiguity × Region interaction in the pooled analysis of all four ROIs, Huynh-Feldt corrected *F*(2.77, 74.79) = 3.24, *p* < 0.05.

Overall, the results from Run1 conformed to our predictions in showing a garden-path effect in the frontal (left pars opercularis) but not the temporal regions (left MTG). Whole-brain analysis of the interaction between Ambiguity and Structure did not reveal any suprathreshold clusters (cluster *p* < 0.05, *z* > 3.1). Uncorrected results (*z* > 3.1) showed activation in the left lateral and medial frontal cortices only (Supplementary Figure 2 in the online supporting information), consistent with our prediction that frontal regions might be the most relevant. Not all frontal regions showed an effect, however, a point we turn to in the Discussion section. All subsequent analyses were restricted to the regions showing an effect.

#### P.2: Change in the RC ambiguity effect over Run2

**Figure 3.**
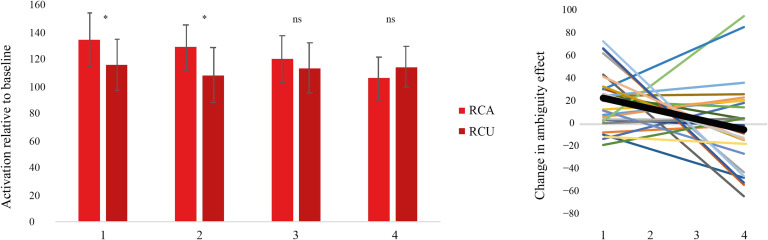
Left: Activation for relative clause ambiguous (RCA) and unambiguous (RCU) structures within the left pars opercularis in Run2. There was an ambiguity effect at the first and second but not at the third and fourth occurrence of verbs. Right: Slope of change over time for each participant. Bold line indicates mean slope.

In Run2, we predicted that the RC ambiguity effect would decrease with exposure to RC sentences, resulting in a negative slope. Within the left pars opercularis, the RC ambiguity effect was significant at the first, *F*(1, 27) = 12.06, *p* < 0.05, and second, *F*(1, 27) = 12.42, *p* < 0.05, but not the third occurrence, *F*(1, 27) = 0.72, *p* > .05, of the verbs. By the fourth occurrence, RC Ambiguous sentences showed numerically lower activation than RC Unambiguous sentences, but this effect was not significant, *F*(1, 27) = 1.59, *p* > 0.05 (Figure 3 left panel). To specifically evaluate whether the RC ambiguity effect decreased with exposure, we performed a linear regression of RC Ambiguous minus RC Unambiguous over four time points and extracted the slope for each participant (Figure 3 right panel). The mean slope was −9.45, *t*(27) = −2.73, *p* < 0.05. Seventeen out of twenty-eight participants had a negative slope (Binomial *p* > 0.05), suggesting some variability (see also section P.3 below).

#### P.3: The relationship between change over Run2 and cognitive control

**Figure 4.**
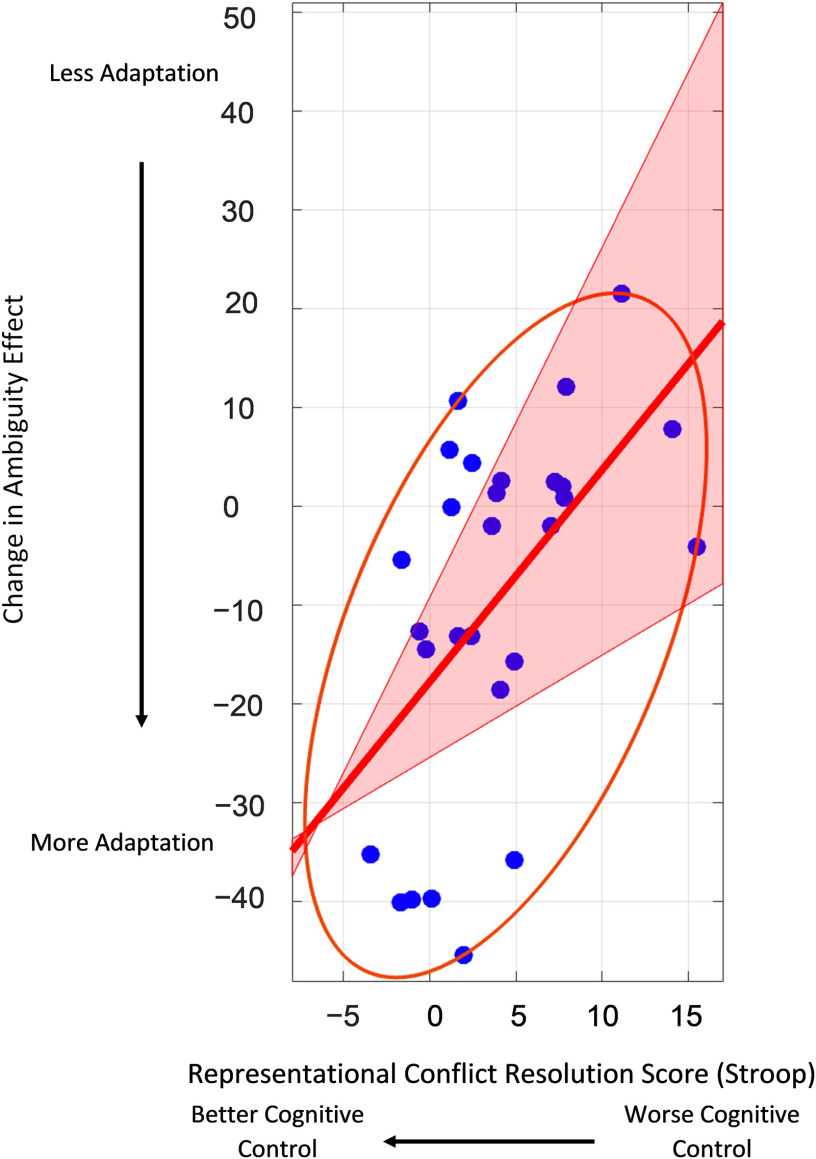
Correlation between individuals’ representational conflict processing score (Stroop) and adaptation of the relative clause ambiguity effect in the left pars opercularis in Run2. Individuals with better cognitive control demonstrated greater adaptation. The ellipse represents the bounding area for included versus excluded bivariate data points. The shaded region represents the 95% CI for the correlation.

We computed Skipped Pearson correlations to evaluate the relationship between the decrease in the ambiguity effect over Run2 and individual differences in cognitive control (Stroop scores). In the left pars opercularis, we observed a significant positive correlation as predicted (Skipped Pearson *r* = 0.544779, CI = [0.298778, 0.739922]). Those who had lower Stroop scores (indicating better cognitive control) were more likely to show a decreasing ambiguity effect with exposure, consistent with our hypothesis (Figure 4).

The association observed above was computed using a robust correlation method that evaluates and accounts for bivariate outliers (Pernet et al., 2013). In addition, we evaluated the reliability of the measurements. For the Stroop conflict score, odd–even split-half reliability (calculated using the Spearman–Brown prophesy formula) was 0.71, suggesting that the measurement was reliable. For the decrease in ambiguity with exposure, we supplemented the original analysis of the slope by calculating difference scores separately using the first and third appearances (third minus first) and the second and fourth appearances (fourth minus second). The two difference scores computed from different subsets of trials were significantly correlated with one another (Skipped Pearson *r* = 0.605445, CI = [0.227441, 0.803849]). Each difference score was also correlated with the Stroop conflict score, consistent with the original analysis (third minus first: *r* = 0.492034, CI = [0.243332, 0.733144]; fourth minus second: *r* = 0.527813, CI = [0.257456, 0.711858]).

#### P.4: Garden-path effects for exposed and unexposed verbs in Run3

**Figure 5.**
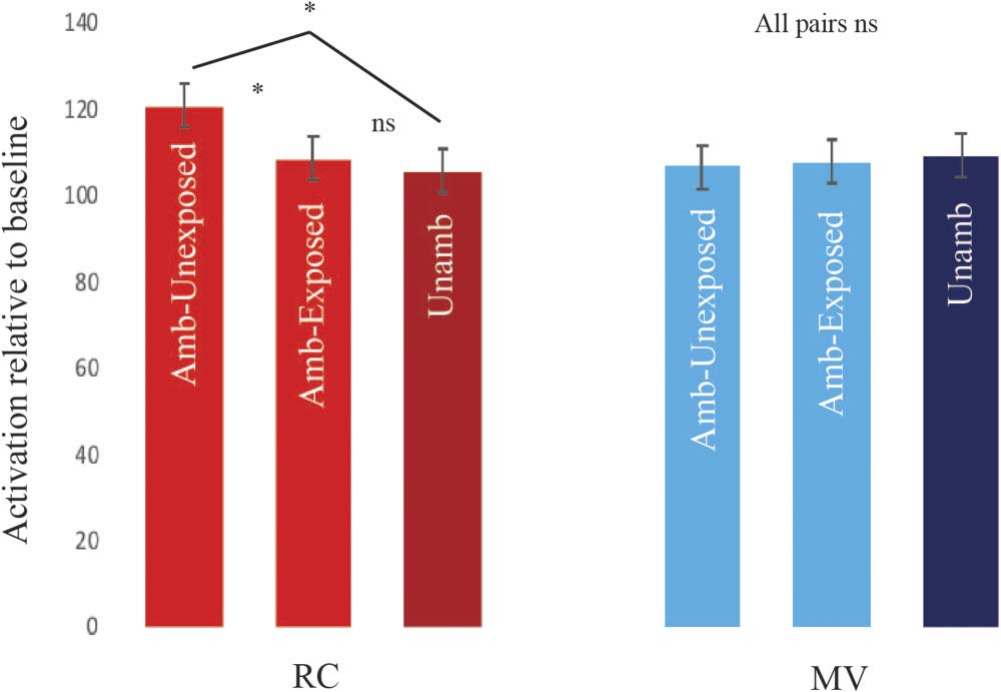
Activation for different sentences relative to baseline in Run3 within the left pars opercularis. Exposed verbs did not show an RC ambiguity effect. Unexposed verbs showed a significant RC ambiguity effect and a difference from exposed verbs. RC = relative clause, MV = main verb, amb = ambiguous, unamb = unambiguous.

In Run3, we asked whether the effect of exposure in the left pars opercularis persisted from Run2 and if so, whether it generalized to verbs other than those that appeared during the exposure phase. For exposed verbs, there was no significant difference between the RC Ambiguous and RC Unambiguous conditions and no interaction between ambiguity and structure (*p*’s > 0.05). In contrast, unexposed verbs showed a significant RC ambiguity effect, *F*(1, 27 = 16.88, *p* < 0.05, and an interaction between ambiguity and structure, *F*(1, 27) = 13.73, *p* < 0.05. Direct comparison between RC Ambiguous Unexposed and RC Ambiguous Exposed revealed a significant difference, *F*(1, 27) = 9.09, *p* < 0.05. In sum, the left pars opercularis showed persistent effects of RC exposure but only for verbs seen repeatedly in that structure (Figure 5).

### Results for the Multiple Demand Network

**Figure 6.**
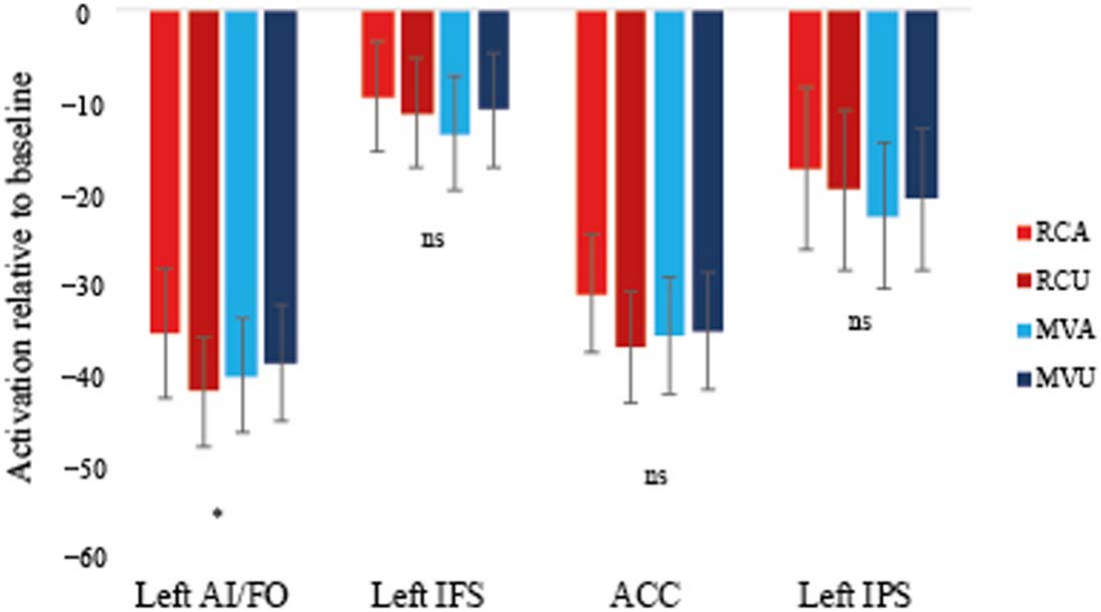
Activation for different sentence types relative to baseline in Run1 within the multiple-demand regions of interest. Only the left AI/FO showed an interaction corresponding to a conflict effect. AI/FO = anterior insula/frontal operculum, IFS = inferior frontal sulcus, ACC = anterior cingulate cortex, IPS = intraparietal sulcus, RCA = relative clause ambiguous, RCU = relative clause unambiguous, MVA = main verb ambiguous, MVA = main verb unambiguous.

We conducted parallel analyses in the MD network as for P.1 to P.4 above. Among the 10 MD ROIs, only the left AI/FO showed a significant interaction between structure and ambiguity in Run1, *F*(1, 27) = 5.48, *p* < 0.05. This interaction was due to a significant effect of ambiguity for RC, RC Ambiguous > RC Unambiguous; *F*(1, 27) = 7.44, *p* < 0.05, but not MV, *F*(1, 27) = 0.32, *p* > 0.05, structures. No other MD region showed this conflict effect. (Please see the Supplementary Information in the online supporting information for details.) Figure 6 shows the pattern in the left AI/FO and for comparison, the patterns in two other frontal regions (left IFS, ACC) and a parietal region (left IPS).

**Figure 7.**
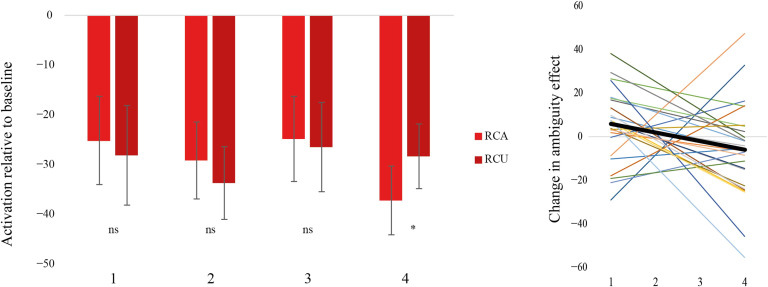
Left: Activation for relative clause ambiguous (RCA) and unambiguous (RCU) structures within left AI/FO in Run2. There was no ambiguity effect at the first to third occurrences and a reverse ambiguity effect at the fourth occurrence of verbs. Right: Slope of change over time for each participant. Bold line indicates mean slope. AI/FO = anterior insula/frontal operculum.

In Run2, within the left AI/FO, there was no ambiguity effect at the first, *F*(1, 27) = 0.51, *p* > 0.05, second, *F*(1, 27) = 1.57, *p* > 0.05, and third, *F*(1, 27) = 0.11, *p* > 0.05, occurrence of verbs. By the fourth occurrence, there was a reverse ambiguity effect such that RC Ambiguous sentences showed lower activation than RC Unambiguous sentences, *F*(1, 27) = 4.59, *p* < 0.05 (Figure 7 left panel). The mean slope was −3.83, *t*(27) = −2.04, *p* = 0.05. Twenty out of twenty-eight participants had a negative slope (Binomial *p* < 0.05; Figure 7 right panel). However, in contrast to the left pars opercularis, there was no correlation between the change in activation in the left AI/FO and Stroop performance (Skipped Pearson *r* = 0.103193, CI = [−0.331871, 0.516918]).

**Figure 8.**
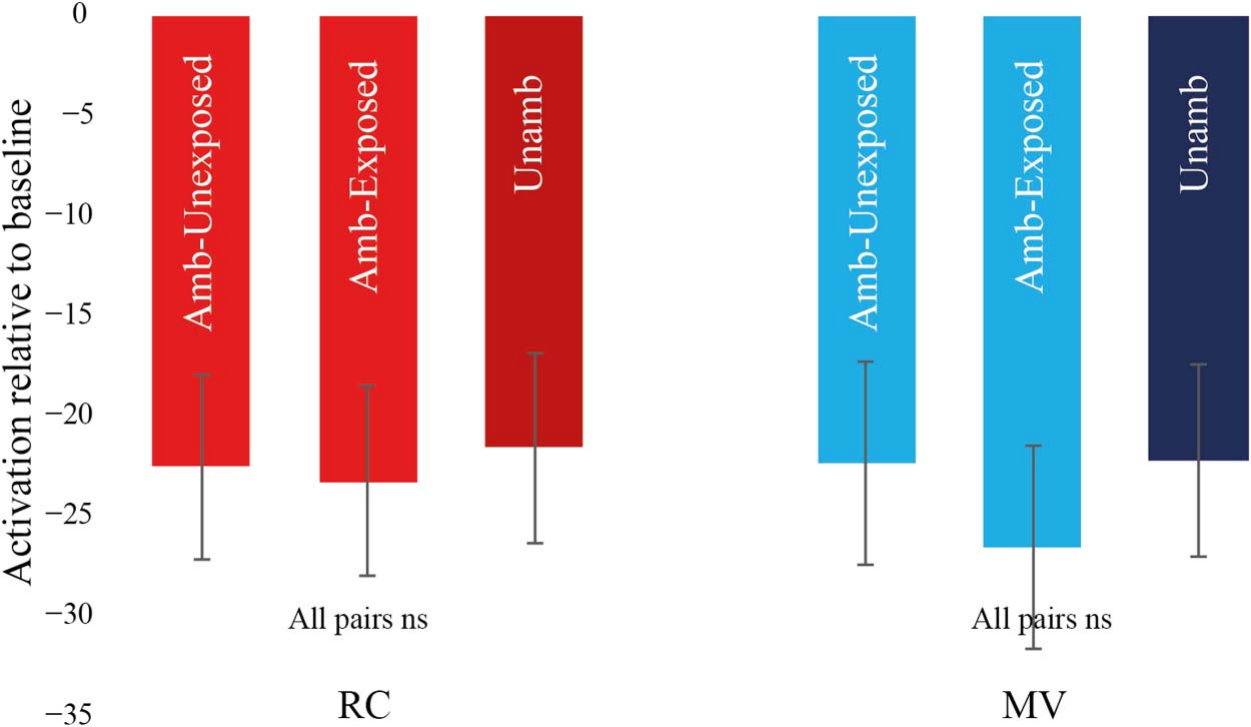
Activation for different sentences relative to baseline in Run3 within the left anterior insula/frontal operculum. Neither exposed nor unexposed verbs showed an RC ambiguity effect. RC = relative clause, MV = main verb, amb = ambiguous, unamb = unambiguous.

In Run3, analyses of exposed and unexposed verbs revealed no RC ambiguity effect or interaction between ambiguity and structure for either verb type. The two conditions also did not differ from one another (all *p*’s > 0.05). Thus, unlike the left pars opercularis, the left AI/FO showed a generalized effect of exposure, which extended to verbs not seen repeatedly in the RC structure (Figure 8).

### Behavioral Results

Accuracy on the comprehension questions was significantly above chance (50%) in all three runs, indicating that participants paid attention to the task and encoded the content of the sentences (Run1: 81.3%, Run2: 77.9%, Run3: 73.6%. One-sample *t* test all *p*’s < 0.05). In Run1, for the length-adjusted RT analyses, the mixed model contained the fixed effects of Ambiguity, Structure, and their interaction along with random intercepts by participant and verb, and random slopes of ambiguity and structure by participant. The results revealed a significant main effect of Structure (β = −123.59, *p* < 0.05) and a significant interaction between Ambiguity and Structure (β = 292.55, *p* < 0.05). The interaction was due to a significant ambiguity effect for RC (Unambiguous *M* = −99.11 ms, Ambiguous *M* = 166.01 ms; β = 263.31, *p* < 0.05) but not MV sentences (Unambiguous *M* = 27.04 ms, Ambiguous *M* = 0.49 ms; β = −26.12, *p* > 0.05). This is consistent with garden-pathing, but strong conclusions might be precluded by the coarse-grained nature of the measure (see more in the Discussion section).

In Run2, we found a significant ambiguity effect at the first (Unambiguous *M* = 13.47 ms, Ambiguous *M* = 174.65 ms; β = 161.08, *p* < 0.05), second (Unambiguous *M* = −47.19 ms, Ambiguous *M* = 166.11 ms; β = 221.33, *p* < 0.05), and fourth occurrence (Unambiguous *M* = −136.94 ms, Ambiguous *M* = 12.31 ms; β = 151.04, *p* < 0.05) of the verbs. There was no effect at the third occurrence (Unambiguous *M* = −68.90 ms, Ambiguous *M* = 15.73 ms; β = 87.46, *p* > 0.05). The mean slope across participants was −17.74, *t*(27) = −0.92, *p* > 0.05. Half the participants showed a negative slope and the other half a positive slope, indicating no consistent pattern. Thus, unlike neural activation in the left pars opercularis and the left AI/FO, behavioral analysis of whole-sentence reading times did not reveal a consistent decrease in the ambiguity effect over RC exposure. The slope of change for the behavioral scores did not correlate with the slope for neural activation within the left pars opercularis (Skipped Pearson *r* = 0.0586045, CI = [−0.25037, 0.384576]) or the left AI/FO (Skipped Pearson *r* = −0.0229688, CI = [−0.492274, 0.445291]).

In Run3, for exposed verbs, the analysis revealed no main effects and no interaction between ambiguity and structure (RC Unambiguous *M* = −45.78 ms, RC Ambiguous *M* = 60.49 ms, MV Unambiguous *M* = 18.48 ms, MV Ambiguous *M* = 12.35 ms; *p*’s > 0.05). In contrast, for unexposed verbs, there was a significant interaction (β = 151.70, *p* < 0.05), which arose from a significant ambiguity effect for RC (Unambiguous *M* = −45.78 ms, Ambiguous *M* = 129.31 ms; β = 175.77, *p* < 0.05) but not MV (Unambiguous *M* = 18.48 ms, Ambiguous *M* = 42.12 ms; β = 22.63, *p* > 0.05). Direct comparison between RC Ambiguous Unexposed and RC Ambiguous Exposed did not reveal a significant difference (*p* > 0.05).

## DISCUSSION

Adults adapt to new input in their native language. To our knowledge, the present study is among the first to investigate the neural mechanisms underlying this adjustment. We exposed participants to a relatively infrequent structure in English that involves RCs and asked if and how their brains adapted to the exposure. Prior to the experimentally manipulated exposure phase, two frontal brain regions—the left pars opercularis and the left AI/FO—showed sensitivity to conflict during sentence processing, namely increased activation for processing ambiguous sentences that resolved to the more infrequent RC structure. During the exposure phase, both regions showed decreasing RC-ambiguity-related activation, suggesting that they were adapting in some way to the current language environment. Following the exposure phase, the adjustment persisted for verbs that had repeatedly appeared in RC structures. These results conformed to our prediction that frontal brain regions would be relevant for adjusting to a novel language environment, consistent with their involvement more broadly in adaptive behavior.

Beyond this general conclusion, the study revealed three contrastive patterns between the two regions that are informative for theorizing about the nature of syntactic updating and the underlying neural substrates: (1) The left pars opercularis ROI, selected using this functional criterion in the first run, was consistently activated for processing sentences compared to baseline in all runs. In contrast, the left AI/FO ROI, which is a part of the MD network, consistently showed the reverse (Baseline > Sentence) pattern. (2) Individual differences in a cognitive control task (Stroop) correlated with adaptation of neural activation with RC exposure within the left pars opercularis but not the left AI/FO. (3) The adaptation effect within the left pars opercularis was verb-specific, showing a difference in adjustment between verbs that had repeatedly appeared in RC sentences during exposure and those that had not. In contrast, the left AI/FO showed broader adaptation and no difference between exposed and unexposed verbs.

The findings for the left pars opercularis are consistent with our proposal that regions within the left inferior frontal cortex could support syntactic updating via their role in handling conflict between incompatible representations. This region showed the expected conflict effect prior to exposure, adaptation during the exposure phase, correlation of adaptation with individual differences in Stroop, and a theoretically meaningful relationship between exposure and adaptation in the form of verb-specificity. By comparison, the results for the left AI/FO suggested involvement in adjusting to a novel environment, but this role appeared to be broad and not specifically related to sentence or conflict processing. This region showed greater activation for a nonsentence task than for sentence reading, no correlation between adaptation and Stroop, and broad adjustment rather than tuning to relevant statistical properties of the language input. Thus, although both regions appear to play a role in adjusting to new language environments, their functions could be importantly different. Before we expand on this topic, we first consider alternative explanations for the observed effects.

### Alternative Explanations

One alternative explanation suggested in the behavioral literature is that adaptation could be happening at the level of generic task practice rather than at the level of updating the likelihood of particular sentence structures. This practice effect could be stronger for the more difficult, ambiguous sentences leading to the observed decrease in the difference between the ambiguous and unambiguous conditions (Prasad & Linzen, 2019; Stack et al., 2018). Neurally, increased facility with the task could correspond to decreased difficulty and, correspondingly, decreased activation in regions that are associated with general cognitive demands. In the context of the current study, this explanation could potentially explain the findings from the left AI/FO but not the left pars opercularis. In the former case, decreased recruitment as the experiment progressed was generic and not restricted to the verbs that were exposed in the RC structures. Thus, it is possible—maybe even likely given the profile of the MD network—that this region broadly supported adjustment to task demands within the context of the experiment. In the left pars opercularis, however, adjustment over the course of the experiment was restricted to those verbs that were seen repeatedly in the RC structures. Thus, this region’s engagement depended on how often individual verbs appeared in a particular structure—a known signature of the sentence processing system. In sum, we suggest that a task-level effect accords well with prior literature on the MD network and the observed results in the left AI/FO but that the left pars opercularis performs a different role (see more below).

Could participants have shifted to a shallower or less attentive (e.g., good-enough) mode of processing for the critical RC Ambiguous sentences in Run3 compared to Run1, which could potentially explain the pattern of lowered ambiguity-related activation as the experiment progressed? Two aspects of the results suggest that this cannot explain the critical findings within the left pars opercularis. First, we found verb-specific adaptation in the neural activation for RC ambiguity in Run3. A general shift in processing mode would be expected to affect all rather than a hypothesized subset of the RC Ambiguous sentences. Second, positive correlation between the decrease in the ambiguity effect and Stroop scores in Run2 follows parsimoniously from the proposed hypothesis about cognitive control and syntactic updating (which in turn was inspired by much prior evidence that cognitive control plays a role in resolving syntactic conflict). In contrast, an explanation based on processing mode would be ad hoc—it is unclear why individuals with better cognitive control should be more likely to shift to good-enough processing over time compared to those with poorer cognitive control. In sum, while we leave open the question of potential shifting between processing modes to future studies with targeted behavioral measures, the activation patterns found in the left pars opercularis are more consistent with the proposed hypothesis. Note that the behavioral measures employed in the present study were mainly intended to verify task compliance and cannot be used to answer this question. Comprehension questions did not focus on the ambiguity and therefore cannot help establish whether participants processed the ambiguity shallowly or deeply. The questions were also not matched or counterbalanced in any specific way (e.g., difficulty) between the two runs. More broadly, whether offline comprehension accuracy can be used to infer the depth of processing of garden-path sentences is not clear (Qian, Garnsey, & Christianson, 2018). Reaction times are expected to get faster whether due to task-level practice, increased experience with a syntactic structure, or a shift in processing mode. These factors could also create asymmetric rather than equal effects in different sentence conditions. Targeted design and analyses are therefore needed to tease apart the different factors and interpret the behavioral measures appropriately.

Could the decreasing RC ambiguity effect in the left pars opercularis be a consequence of short-term syntactic priming? We think this is unlikely for two reasons. First, we show that the diminished RC ambiguity effect for the exposed verbs persisted from the end of Run2 going into Run3. This gap of several minutes is not consistent with purely short-term priming. Second, growing consensus within the syntactic priming literature eschews dichotomy between priming and longer-term adjustment. Because priming lasts across multiple intervening trials and shows signatures of implicit learning (Bock & Griffin, 2000; Fine & Jaeger, 2013; Wells et al., 2009), the dominant current view is that (long-term) syntactic priming is syntactic updating. The present findings are consistent with results that have implicated the left inferior frontal cortex in adaptation within a priming context (Segaert, Kempen, et al., 2013; Segaert, Menenti, et al., 2011). Future studies comparing neural adaptation at different time lags following exposure could be useful for additionally clarifying the underlying mechanism(s).

### The Role of the Left Pars Opercularis

Some researchers have suggested that the left pars opercularis (and Broca’s area more broadly) is responsible for specific operations (e.g., movement) that are associated with syntactic complexity (e.g., Ben-Shachar et al., 2003). The current results do not fit within this perspective because (a) RC Unambiguous sentences are just as complex as RC Ambiguous sentences, but we detected higher activation for the former in Run1; (b) the complexity of these sentence types and their associated operations do not change with exposure, but activation with the left pars opercularis did; and (c) the operations do not differ based on the verb, but the observed results showed a difference between exposed and unexposed verbs.

Prior evidence on the left inferior frontal cortex and syntactic ambiguity and conflict processing are more directly relevant to the present findings (January et al., 2009; Mason et al., 2003; Rodd, Longe, et al., 2010). We extend previous results by showing that the involvement of the left pars opercularis for conflict-inducing sentences adapts with additional exposure. Thus, this region is engaged in updating syntactic expectations in individuals’ native language. The observed patterns are broadly consistent with our description of this region’s role in terms of cognitive control. In line with current thinking on how cognitive control operates, we would suggest that the left pars opercularis is involved in biasing the activation of and resolving the conflict between linguistic representations that reside elsewhere in posterior cortical regions (January et al., 2009; Novick et al., 2005; Thothathiri et al., 2012; Ye & Zhou, 2009). In the context of the present study, this would mean adjusting the activation levels of RC and MV structures such that the less frequent RC representation wins out over the more prepotent MV representation when applicable. Such a function would be most needed when the parser encounters ambiguous sentences that resolve towards the less frequent RC structure, with that need diminishing upon increasing exposure to that structure. Below we discuss nuanced questions within this broad perspective.

One question pertains to the domain-specificity or generality of this region’s function. On one end of the spectrum, some have proposed that this region could be engaged in domain-general cognitive control across linguistic and nonlinguistic tasks (e.g., Hsu et al., 2017; January et al., 2009). Others have suggested that different subregions within the left frontal cortex could be specialized for the controlled processing of different types of representations (e.g., Hagoort, 2016). The present study did not test a variety of linguistic and nonlinguistic tasks in the scanner and cannot address this question directly. Correlation between adaptation within the left pars opercularis and individuals’ behavioral performance on Stroop (a nonsyntactic task) suggests that this region’s role is not specific to syntax. This is consistent with prior evidence for the co-localization of sentential and nonsentential tasks within the left frontal cortex (January et al., 2009; van de Meerendonk, Rueschemeyer, & Kolk, 2013; Ye & Zhou, 2009). Such an interpretation leaves open the possibility that this region is engaged for language-specific rather than syntax-specific representations (because the Stroop task involved linguistic content). However, because adjacent regions within the frontal cortex can have different processing profiles (Fedorenko et al., 2013; Hagoort, 2016), we cannot definitively rule out specialization for syntax. We observed the expected patterns in the left pars opercularis but not the adjacent pars triangularis or the frontal orbital cortex ROIs, which is potentially consistent with the suggestion that the posterior parts of the left inferior frontal cortex might be specialized for the controlled syntactic processing (Hagoort, 2016). For the present, we suggest that this region could be involved in either syntactic updating or broader language updating.

A different question pertains to the processing stage at which the left pars opercularis is involved in updating. This region could help resolve conflict between competing representations thereby influencing the updating process (as we have suggested). Alternatively, activation within this region could provide a read-out of prediction error, which could serve as input for updating that recruits some other brain region. Both interpretations are consistent with the observed results—both prediction error and the need for conflict resolution would be high for RC Ambiguous sentences in Run1 because this structure is less expected, both would decrease over time as the parser updates the probability of RC structures, and both could depend on characteristics that affect syntactic expectations (e.g., verb-structure links). Prior literature on cognitive control suggests that the lateral prefrontal cortex broadly is involved in conflict resolution. In contexts that do not involve updating or prediction error (e.g., Stroop), higher activation within these regions is associated with improved behavioral performance on a given trial (Kerns, 2006; Kerns et al., 2004). However, definitive evidence might require causal paradigms with sufficient temporal resolution to determine if and how updating is disrupted without a functioning left pars opercularis.

### The Role of the Left Anterior Insula/Frontal Operculum

Evidence from resting state functional connectivity studies suggests that the left AI/FO is part of a cingulo-opercular network that is involved in task set maintenance and attentional modulation (Dosenbach, Fair, et al., 2007). This region is recruited at the beginning of a given task block and remains active for the duration of a task, suggesting that it is involved in initiating and maintaining a task set. Critically, these activation patterns are found across multiple domains, providing evidence for a domain-general function in goal-directed behavior (Dosenbach, Visscher, et al., 2006). The left AI/FO is part of the MD network, which shows increased recruitment for difficult tasks and the representation of task-relevant over irrelevant information (Woolgar, Williams, & Rich, 2015). Together, this evidence has led researchers to propose that this region could provide domain-general task-level and attentional support to the processing of domain-specific information that occurs in other more specialized regions (Dosenbach, Visscher, et al., 2006; Woolgar et al., 2015).

The results from the present study are consistent with prior evidence and interpretation. In particular, the left AI/FO (along with other MD regions) showed greater activation during the baseline task than during sentence reading across all three runs. The former task involved false fonts and was more novel to participants than the latter (reading being a familiar activity to our literate participants). During practice prior to scanning, participants had a mean accuracy of 65.7% and 97.7% on the baseline and sentence reading tasks, respectively. This suggests that the baseline task was more difficult in some respects. Engagement of the AI/FO could have supported the maintenance of task sets, especially in the context of different interwoven tasks like in the present study. As reviewed above, this function is expected to be sensitive to task difficulty (explaining greater recruitment for the baseline over the sentence task, and for harder over easier sentences) and domain-general. The adaptation of left AI/FO activation in Run2 and the persistence of this adaptation for both unexposed and exposed verbs in Run3 is consistent with such a task-set maintenance function. As the experiment progressed and different tasks and sentence types became more familiar, this region might have become less and less needed.

Why did we see sensitivity to sentence processing difficulty within the left AI/FO and no other frontal MD regions? It is worth reiterating here that we used MD network ROIs based on group-level coordinates from the prior literature. This could have potentially obscured patterns that might be better detected using methods (e.g., subject-specific localizers) that take variability between individuals into account. Given this caveat, we can only speculate post hoc about the observed pattern. It is possible that frontal MD regions (left and right IFS) that are a part of a frontoparietal network that usually shows short-lived activity in response to a cue (cf. sustained activity in the cingulo-opercular network; Dosenbach, Fair, et al., 2007) were not as relevant in the task contexts of the present study. Alternatively, sustained activation differences between harder versus easier tasks might have been easier to detect than short-lived activation differences. More challenging to explain is why other regions within the cingulo-opercular network—namely the right AI/FO and the ACC—did not show sensitivity to sentence processing difficulty like the left AI/FO. For the former, fine-grained functional specialization within AI/FO regions is still an open question (Ullsperger, Harsay, Wessel & Ridderinkhof, 2010). With respect to the ACC, this region has been linked to response rather than representational conflict and might be more likely to be engaged in tasks requiring overt responses (Milham et al., 2001; Thothathiri, 2018) unlike sentence comprehension. Alternatively, the ACC might be especially important for comprehension in a social pragmatic context (Hagoort, 2016). Future studies are needed to tease apart the roles of different cingulo-opercular regions within the MD network during sentence comprehension. Broadly, all of these roles are expected to be domain-general and sensitive to task difficulty.

### Broader Implications and Future Directions

While the current study addresses syntactic updating, processing ambiguity and updating expectations also applies at other linguistic levels, including lexical semantics. Models of how the parser addresses lexical-semantic ambiguity mirror models of syntactic processing. Specifically, multiple possible meanings for a given word are activated simultaneously and these meanings have an associated probability based on frequency of use. The most frequent meaning is typically preferred (e.g., Twilley & Dixon, 2000). However, when semantically ambiguous words are repeatedly encountered in contexts that support the less frequent meaning, individuals are more likely to activate the less likely meaning in later probes (Gilbert, Davis, Gaskell, & Rodd, 2018; Rodd et al., 2016; Rodd, Cutrin, Kirsch, Millar, & Davis, 2013). Synonyms for the ambiguous word do not show this effect, indicating updating of form–meaning mappings rather than semantic priming (Rodd et al., 2013). The effects build up over time, persist for 20 min or more, and are sensitive to surprisal (Gilbert et al., 2018; Rodd et al., 2013). These parallels to syntactic processing suggest that the current hypothesis might also apply to lexical-semantic updating. Namely, cognitive control could help disambiguate the meaning of ambiguous words and thereby assist in updating the likelihood of less frequently encountered meanings. Regions within the left inferior frontal cortex and the MD network could play a role in such adaptation, possibly with adjacent subregions showing specialization for different linguistic processes and/or different patterns of connectivity with posterior cortices. Thus, comparing different kinds of updating within language could be a fruitful future direction for testing the hypothesis proposed here in a closely related domain and for clarifying the networks that support different aspects of language.

The results of the present study are from written comprehension. Previous evidence suggests that activation in frontotemporal language regions during sentence comprehension is similar between the visual and auditory modalities (Devauchelle et al., 2009; Jobard, Vigneau, Mazoyer, & Tzourio-Mazoyer, 2007). Further, P600 signals in response to syntactic expectation violations are also similar between the two modalities (Hagoort & Brown, 2000). Therefore, we expect the conclusions from the current study to apply broadly to sentence comprehension (written or spoken). However, spoken language contains additional information, especially prosodic cues, which could guide online syntactic expectations. Future studies could investigate whether the presence or absence of disambiguating prosodic cues alters the need for cognitive control and the recruitment of different brain regions during syntactic updating.

Previous behavioral studies have shown that adults can adjust their syntactic expectations based on the particular speaker (Kamide, 2012; Kroczek & Gunter, 2017). One interesting extension of the current paradigm might be to investigate the neural substrates that support switching between different sets of syntactic expectations for different speakers. A priori, control regions that are known to support task-switching or set-shifting might be expected to play a role in such situations (Dove, Pollmann, Schubert, Wiggins, & Von Cramon, 2000; Swainson et al., 2003; Ruge, Jamadar, Zimmermann, & Karayanidis, 2013). Further, Kroczek and Gunter (2017) showed that participants maintained some information about speaker-specific syntactic probabilities over the course of months. However, this was not immediately deployed (say from memory) but re-established quickly with limited renewed exposure to the speakers. The cues that trigger quick adjustment and the neural mechanisms that support it and any potential transfer of learning from one context to another are open questions for future investigation.

The results from the present study could be strengthened in a number of ways. First, we examined regions linked to cognitive control because we wanted to test a particular process-specific hypothesis. We would not claim, however, that those are the only regions relevant to updating. In this context, it is worth discussing the null conflict effect in Run1 for the MTG, which appears to stand in contrast to previous findings of syntactic and lexical ambiguity effects in the posterior temporal cortex (e.g., Mason et al., 2003; Rodd, Longe, et al., 2010). The divergent outcomes could potentially be related to methodological differences. For example, Mason et al. (2003) examined a temporal ROI that was defined anatomically and included posterior, superior, and middle temporal areas whereas the current study examined a functionally defined MTG region. Rodd, Longe, et al. (2010) investigated a different kind of syntactic ambiguity than was studied here. Regardless, like many other researchers, we would suggest that left frontal and temporal areas work in close concert during sentence processing. The lack of an effect in the left MTG (in contrast to the left pars opercularis) could have resulted from lack of power due to analyzing a single run and/or the type and strength of the conflict manipulation. It is possible that alternative paradigms with more sensitivity will detect effects in both left frontal and temporal areas. Further research is needed to fully establish whether these regions play related but distinct roles (e.g., see the discussion in Mason et al., 2003 and Rodd, Longe, et al., 2010) during syntactic adaptation. Future studies could also explore the neural substrates related to other components such as the encoding of syntactic probabilities and the connectivity between different brain regions that facilitates updating as a whole.

Second, our Run3 results showed no RC ambiguity effect for verbs that were frequently experienced in those structures. For the left pars opercularis, this pattern was verb-specific and did not extend to unexposed verbs, which we interpreted as demonstrating a specific effect of RC exposure rather than a generic effect of task practice. Future studies could explicitly manipulate exposure type (e.g., RC sentences versus other kinds of fillers; see Prasad & Linzen, 2019; Stack et al., 2018) in order to establish more definitively whether it is repeated experience with RC structures in particular that causes neural adaptation. Finally, replication of the Stroop correlation in a larger sample, differential correlations with different cognitive control tasks, and concomitant neuroimaging of multiple language and cognitive control tasks would allow for stronger inferences about the particular aspects of cognitive control that are related to syntactic updating and the extent to which those processes are domain-specific or general.

In conclusion, emerging research indicates that adults, not just children, continue to update their native language based on new experiences. The current study shows that left frontal cortical regions implicated in cognitive control play a role in updating syntactic expectations. The utility of these regions could encompass broad support for attentional demands and task maintenance during sentence processing as well as more specific resolution of conflict that arises from encountering unexpected sentences. Together, these areas could help ensure that updating is accurate and efficient, thereby enabling language users to better navigate diverse and variable language environments.

## ACKNOWLEDGMENTS

We would like to thank Maria Braiuca for her assistance in data collection. We would also like to thank Dr. John Van Meter and the research assistants at the Center for Functional and Molecular Imaging (CFMI) at Georgetown University.

## FUNDING INFORMATION

This study was funded in part by a University Facilitating Fund grant from the George Washington University to Malathi Thothathiri.

## AUTHOR CONTRIBUTIONS

Kelly Sharer and Malathi Thothathiri designed the experiment. Kelly Sharer created the materials and collected data. Kelly Sharer and Malathi Thothathiri analyzed the data, designed the figures, and wrote the manuscript.
